# Multilevel and spatial analyses of childhood malnutrition in Uganda: examining individual and contextual factors

**DOI:** 10.1038/s41598-020-76856-y

**Published:** 2020-11-18

**Authors:** Prince M. Amegbor, Zhaoxi Zhang, Rikke Dalgaard, Clive E. Sabel

**Affiliations:** 1 Big Data Centre for Environment and Health (BERTHA), Department of Environmental Science, Aarhus University, Frederiksborgvej 399, 4000 Roskilde, Denmark; 2 Department of Environmental Science, Aarhus University, Frederiksborgvej 399, 4000 Roskilde, Denmark

**Keywords:** Environmental impact, Environmental social sciences, Nutrition disorders

## Abstract

In this study, we examine the concepts of spatial dependence and spatial heterogeneity in the effect of macro-level and micro-level factors on stunting among children aged under five in Uganda. We conducted a cross-sectional analysis of 3624 Ugandan children aged under five, using data from the 2016 Ugandan Demographic and Health Survey. Multilevel mixed-effect analysis, spatial regression methods and multi-scale geographically weight regression (MGWR) analysis were employed to examine the association between our predictors and stunting as well as to analyse spatial dependence and variability in the association. Approximately 28% of children were stunted. In the multilevel analysis, the effect of drought, diurnal temperature and livestock per km^2^ on stunting was modified by child, parent and household factors. Likewise, the contextual factors had a modifiable effect on the association between child’s sex, mother’s education and stunting. The results of the spatial regression models indicate a significant spatial error dependence in the residuals. The MGWR suggests rainfall and diurnal temperature had spatial varying associations with stunting. The spatial heterogeneity of rainfall and diurnal temperature as predictors of stunting suggest some areas in Uganda might be more sensitive to variability in these climatic conditions in relation to stunting than others.

## Introduction

It is widely acknowledged that climate change poses a significant threat to the health and wellbeing of poor and vulnerable populations in this century^1–3^. The research shows that global climate change will have a significant impact on food production and food security, especially in the developing world^4–6^. Agriculture and food production in many developing countries, including those in sub-Saharan Africa, are dependent on local climatic factors^7,8^. The majority of individuals and households in sub-Saharan Africa depend on rain-fed agriculture for their sustenance and their nutritional requirement^2,9^. This traditional form of food production results in low yields and has a very low adaptive capacity to climate variabilities^10–13^. In view of this, many researchers and stakeholders suggest climate change poses a significant threat to the attainment of the sustainable development goal of ending hunger and all forms of malnutrition in sub-Saharan Africa^14–16^. Among other factors, the region’s susceptibility to climate variability and low agricultural productivity have been acknowledged major contributors to the high prevalence of persistent hunger among its population^3,17,18^.

Sub-Saharan Africa has the highest global prevalence of hunger with 22.8% prevalence in 2017; representing an increase of 2.5% in 2017 compared to 2014 (20.9%)^17^. Similarly, the number of undernourished people has increased from 195 million people in 2014 to approximately 237 million people in 2017^17^. The burden of malnutrition is significantly higher among children under 5 years of age^19^. Eastern African region, where Uganda is located, has the second highest percentage of stunted children aged under 5 (35.2%) after the Oceania region (38.2%)^20^. Nevertheless, the evidence from existing studies shows that there are significant regional or spatial variations in the prevalence of malnourished children in Uganda and the sub-Saharan African region^2,8,21–23^. In Uganda, the proportions of children suffering persistent malnutrition (stunting) and acute malnutrition in the Kampala region (national capital region), are significantly lower compared to other regions^23^. For instance, 13.5% and 5.7% of children in the Kampala region suffer from persistent and acute malnutrition, respectively, compared to 45.0% and 31.9% of children in the Karamoja region (one of the poorest regions in the country)^24^.

Evidence from the literature and research on childhood malnutrition shows that the causes of childhood malnutrition are multifaceted, emanating from biological, social, cultural, economic, and environmental factors. Childhood malnutrition in sub-Saharan Africa and many parts of the developing world is significantly associated with variability in rainfall and temperature^8,9,16,21,25–27^. Extreme rainfall and drought affect agricultural productivity, especially among subsistence farmers, contributing to food insecurity and malnutrition among children^8,9^. Childhood malnutrition is also strongly associated with poverty, ill-health and human capital^28–30^. Findings of existing studies show that children in socioeconomically disadvantaged households are more susceptible to childhood malnutrition^31–34^. Some researchers argue that variabilities in child development and malnutrition status among social groups and locations are more often due to socioeconomic inequalities than biological factors^35^. Likewise, childhood malnutrition is also associated with major health problems in early childhood and later life^36^. Poor nutrition among children contributes to about 45% or 3.1 million cases of child mortality^30^. The literature shows that malnourished children are more susceptible to cognitive impairment and intellectual disabilities^36,37^. Malnourished children are more likely to perform poorly in academic and psychological assessments. A study in Barbados found that moderate and severe childhood malnutrition significantly elevated the risk of impaired intelligence quotient (IQ); persons with childhood malnourishment history were nine times more likely to have intellectual disability^37^. Adults with a history of childhood malnutrition are also known to be more likely to have personality disorders, such as paranoid, schizoid and dependent personality disorder^36^.

While existing studies have examined the association between climatic factors and childhood malnutrition, their methodological approaches do not address the issue of spatial dependence and spatial heterogeneity in this association. That is, the findings of these studies assume the association between climatic factors and childhood malnutrition is the same or stationary across the study area. Research in other health issues has observed the existence of spatial autocorrelation and clustering in the association between socioeconomic, geographical factors, and major health outcomes^38,39^. Compared to traditional regression models, spatial regression models move beyond the naïve assumption of the association between predictors and outcome being constant across space. They consider the potential effect of neighbouring geographic units on the observed association (spatial autoregressive model) and variability in the association across geographic areas^40^. The knowledge from these spatial models is relevant for identifying policies and intervention areas to reduce the effect of climatic variability on health and wellbeing, especially childhood malnutrition. In this study, we examine spatial variability and spatial dependence in the association between individual factors, contextual factors, and childhood malnutrition among children under five in Uganda. Specifically, we sought to (a) examine the spatial pattern of childhood malnutrition in Uganda (b) determine whether childhood malnutrition is independent or non-independent across districts (counties) in Uganda (c) explore whether there is spatial heterogeneity in the association between socioeconomic characteristics, climatic factors and childhood malnutrition in Uganda.

## Results

### Descriptive

Table 1 shows the descriptive statistics for the study variables. Among children aged under five in Uganda, 28.07% were stunted. The result shows that the mean drought episode in the country is 1.45 units with an average aridity index of 33.13. The mean annual rainfall for the years 2010 and 2015 was 1359.20 mm with an average diurnal temperature of 12.43 and a mean annual temperature of 23.40. The average head of livestock was 82.19 per km^2^. There was almost equal (50:50) sample of female and male children and had an average weight at birth (52.71%). The majority of parents had only a primary level of education; 62.35% for mothers and 54.29% for fathers. The percentage of fathers with secondary and post-secondary education (38.75%) was relatively higher than that of mothers (27.32%) The majority of mothers were employed in the agriculture sector (46.97%) while the majority of fathers were employed in service and manual labour sector (39.70%). A higher proportion of mothers were unemployed (6.96%) compared to fathers (3.43). The majority of households were rural (79.80%) and 43.26% of children lived in the poorest and poorer households. Table 2, shows the summary statistics of the study variables for our spatial models. The mean district percentage of stunted children was 27.95%. The district average of uneducated and unemployed mothers were 13.34% and 15.98%, respectively. The district average for uneducated and unemployed fathers were 9.43% and 3.53%, respectively.

**Table 1:** Descriptive summary of study variables (n = 3624).

	Frequency (percentage)
**Malnutrition indicators**	
Stunting	
Yes	1020 (28.15)
No	2604 (71.85)
**Child characteristics**	
Sex of child	
Male	1800 (49.67)
Female	1824 (50.33)
Weight at birth	
Average	1921 (53.01)
Very large	296 (8.17)
Larger than average	704 (19.43)
Smaller than average	514 (14.18)
Very small	189 (5.22)
**Parental characteristics**	
Level of education—Mother	
No formal education	448 (12.36)
Primary education	2326 (64.18)
Secondary education	661 (18.24)
Post-secondary education	189 (5.22)
Sector of employment—Mother	
Unemployed	623 (17.19)
Agriculture	1711 (47.21)
Service and manual	743 (20.50)
Professional	547 (15.09)
Level of education—father	
No formal education	311 (8.58)
Primary education	2012 (55.52)
Secondary education	921 (25.41)
Post-secondary education	380 (10.49)
Sector of employment—father	
Unemployed	146 (4.03)
Agriculture	1334 (36.81)
Service and manual	1450 (40.01)
Professional	694 (19.15)
**Household characteristics**	
Income index	
Poorest	980 (27.04)
Poorer	775 (21.39)
Middle	698 (19.26)
Richer	635 (17.52)
Richest	536 (14.79)
Household type	
Urban	594 (16.39)
Rural	3030 (83.61)
**Environmental factors**	
Drought episode	1.45 (0.03)^a^
Mean aridity 2015 and 2010	33.13 (0.10)^a^
Mean rainfall 2015 and 2010 (mm)	1359.20 (4.05)^a^
Mean diurnal temperature 2015 and 2010 (°C)	12.43 (0.01)^a^
Mean annual temperature 2015 and 2010 (°C)	23.40 (0.03)^a^
Mean heads of livestock per km^2^	82.19 (1.72)^a^

^a^Mean, standard errors in parenthesis.

**Table 2:** Descriptive of summary of study variables for the spatial models (n = 112 districts).

Variables	Mean	Standard error	95% Confidence interval	Minimum	Maximum
Stunting (%)	27.95	1.33	25.32–30.58	0	82.82
Uneducated mothers (%)	13.34	1.75	9.87–16.82	0	95.3
Unemployed mothers (%)	15.98	1.59	12.84–19.13	0	66.72
Uneducated fathers (%)	9.43	1.71	6.03–12.83	0	98.9
Unemployed fathers (%)	3.53	0.69	2.17–4.89	0	43.27
Poorest and poorer households (%)	49.49	2.75	44.03–54.95	0	100
*Mean heads of livestocks per km*^*2*^	56.12	4.63	46.94–65.30	1.48	380.64
*Mean aridity 2015 and 2010*	32.08	0.51	31.07–33.09	15.53	47.42
*Mean rainfall 2015 and 2010 (mm)*	1344.41	22.14	1300.53–1388.28	724.91	1913.33
*Mean diurnal temperature 2015 and 2010 (°C)*	12.51	0.07	12.36–12.65	10.76	14.2
*Mean annual temperature 2015 and 2010 (°C)*	23.43	0.17	23.11–23.77	17.03	26.53

**Figure 1:**
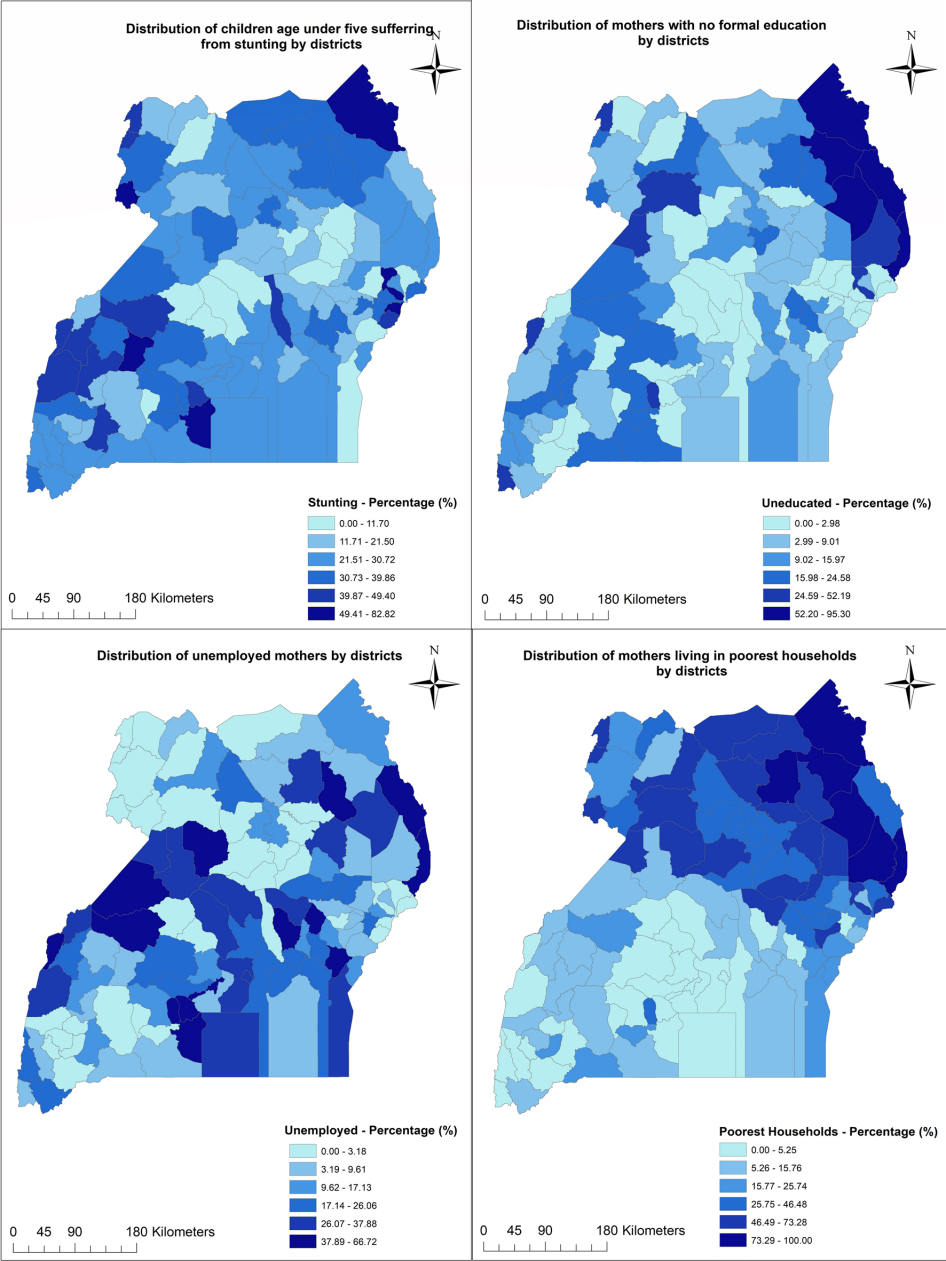
Distribution of stunting, maternal education, maternal unemployment and poor households by districts. Generated with ArcMap 10.6 by ESRI (https://desktop.arcgis.com/en/).

Figure 1 displays the percentage distribution of stunted children, uneducated mothers, unemployed mothers, and poor households in Uganda by districts. The result for stunting suggests the district distribution of stunted children appears random. Out of the 112 districts in Uganda, 51 districts had a stunting rate above the national average of 28.07% as reported in Table 1. The Bududa district located in eastern Uganda had the highest percentage of stunted children—82.82%. The result also shows that the percentage of unemployed mothers is randomly distributed with no distinct clustering pattern. The percentage distribution of uneducated mothers shows distinct clustering in the north-eastern corner of the country. Amudat district in northern Uganda had the highest percentage of uneducated mothers (95.30%). The distribution of poor households also shows clustering in the northern and eastern parts of the country. In the Northern and Eastern regions of the country, the majority of districts had 26% or more households in poor wealth quintile; compared to the Western and Central part where the range of poor households was between 0 and 26%. A total of 48 districts had a higher percentage of uneducated mothers compared to the average reported in Table 1 (10.34%) and 61 districts had a higher percentage of poor households compared to the average proportion of poorest and poorer households reported in Table 1 (43.26%).

### Multilevel mixed-effect analysis

Table 3 displays the result of the multilevel-mixed effect analysis. The bivariate model shows that for climatic factors a degree Celsius increase in the mean annual temperature was significantly associated with a decreased likelihood of stunting among children under age five (expβ = 0.939, *p* < 0.05). A unit increase in the mean head of livestock per km^2^ reduces the likelihood of stunting among children (expβ = 0.999, *p* < 0.05). The child’s sex and weight at birth were significantly associated with stunting. Female children were less likely to be stunted (expβ = 0.853, *p* < 0.05); children whose weight at birth was smaller than average and very small were more likely to be stunted. With regard to parental characteristics, both parents' level of education and mother’s type of employment were significantly associated with stunting. Children of highly educated parents and children whose mothers worked in a professional occupation were less likely to be stunted. Children in richer and richest households were less likely to be stunted; while children in rural households were more likely to be stunted compared to those in urban areas (expβ = 1.403, *p* < 0.01).

**Table 3:** Multilevel mixed-effect analysis of determinants of stunting among children aged under five.

	Bivariate model	Model 1	Model 2	Model 3
**Environmental factors**				
Drought episode	1.056 (0.059)	1.160 (0.142)		1.272 (0.141)*
Mean aridity 2015 and 2010	1.008 (0.010)	0.931 (0.022)**		0.948 (0.026)*
Mean rainfall 2015 and 2010 (mm)	0.999 (0.001)	1.001 (0.001)**		1.001 (0.001)**
Mean diurnal temperature 2015 and 2010 (°C)	0.964 (0.068)	0.833 (0.102)		0.787 (0.086)*
Mean annual temperature 2015 and 2010 (°C)	0.939 (0.026)*	0.685 (0.034)***		0.687 (0.040)***
Mean heads of livestock per km^2^	0.999 (0.001)*	0.999 (0.001)		1.003 (0.001)*
**Child characteristics**				
Sex of child				
Male (ref)				
Female	0.853 (0.066)*		0.811 (0.065)**	0.825 (0.082)
Weight at birth				
Average (ref)				
Very large	0.753 (0.120)		0.747 (0.114)	0.927 (0.151)
Larger than average	0.797 (0.110)		0.767 (0.104)	0.735 (0.138)
Smaller than average	1.293 (0.146)*		1.317 (0.151)*	1.608 (0.246)**
Very small	1.758 (0.293)***		1.859 (0.311)***	1.961 (0.505)**
**Parental characteristics**				
Level of education—Mother				
No formal education (ref)				
Primary education	0.885 (0.108)		0.879 (0.117)	0.849 (0.135)
Secondary education	0.603 (0.085)***		0.714 (0.114)*	0.657 (0.165)
Post-secondary education	0.183 (0.049)***		0.335 (0.104)***	0.673 (0.286)
Sector of employment—Mother				
Unemployed (ref)				
Agriculture	1.019 (0.116)		0.833 (0.099)	0.835 (0.166)
Service and manual	1.064 (0.139)		0.972 (0.135)	0.851 (0.165)
Professional	0.552 (0.097)***		0.711 (0.104)	0.599 (0.155)*
Level of education—father				
No formal education (ref)				
Primary education	1.066 (0.191)		1.158 (0.224)	1.055 (0.298)
Secondary education	0.846 (0.169)		1.081 (0.241)	1.126 (0.335)
Post-secondary education	0.430 (0.118)**		0.852 (0.243)	0.887 (0.350)
Sector of employment—father				
Unemployed (ref)				
Agriculture	1.400 (0.321)		1.538 (0.385)	1.596 (0.528)
Service and manual	1.275 (0.297)		1.536 (0.386)	1.709 (0.539)
Professional	0.880 (0.229)		1.366 (0.379)	1.345 (0.565)
**Household characteristics**				
Income index				
Poorest (ref)				
Poorer	0.887 (0.109)		0.930 (0.116)	0.837 (0.136)
Middle	0.834 (0.097)		0.874 (0.106)	0.811 (0.132)
Richer	0.678 (0.087)**		0.789 (0.109)	0.636 (0.109)**
Richest	0.318 (0.057)***		0.455 (0.118)**	0.440 (0.123)**
Household type				
Urban (ref)				
Rural	1.403 (0.167)**		0.899 (0.131)	1.256 (0.216)
**Random model**				
Variance—districts		1.04e−38 (1.81e−37)	0.173 (0.056)	1.12e−34 (6.30e−34)
Variance—PSU		0.165 (0.080)	0.262 (0.078)	0.179 (0.113)
ICC—districts		5.75e−39	0.083	6.14e−35
ICC—PSU		0.091	0.126	0.098
**Model diagnostics**				
AIC		1642.347	4077.509	1609.684
Wald Chi^2^		64.95***	244.59***	404.99***

Robust standard error in parenthesis; ***p < 0.001; **p < 0.01; *p < 0.05.

The first multivariate model (Model 1) considered the contextual factors only. The result indicates mean aridity, mean annual rainfall, and mean annual temperature were significantly associated with stunting among children under age five. A unit increase in the aridity index (or increase in wetness) reduced the likelihood of stunting among children (expβ = 0.931, *p* < 0.01). A degree Celsius increase in the mean annual temperature reduces the likelihood of stunting among children (expβ = 0.685, *p* < 0.001). However, a millimetre increase in mean annual rainfall increases the likelihood of stunting among children under age five. The intra-class correlation (ICC) estimates indicate that differences in neighbourhood account ~ 9% of the variability in stunting. Model 2 considers child, parents and household factors only. The result shows that female children, children whose mothers have secondary education and above, and children who live in the richest households were less likely to be stunted. Similar to the bivariate model, children who were smaller than average and very small at birth were more likely to be stunted compared to those who were average at birth. Differences in neighbourhoods and districts accounted for ~ 13% and ~ 8%, respectively, of the variability in stunting. In the final multivariate model (Model 3), all contextual factors became statistically significant predictors of stunting among children aged under five. A unit increase in drought episode mean annual rainfall and mean heads of livestock per km^2^ increased the likelihood of stunting among children aged under five. On the other hand, a unit increase in mean aridity, mean diurnal temperature and mean annual temperature reduced the likelihood of stunting among children aged under five. Weight at birth, the mother’s type of occupation and household wealth index were significantly associated with stunting. Children whose weight at birth was smaller than average and very small were more likely to be stunted. Children whose mothers worked in the professional sector were less likely to be stunted compared to those with unemployed mothers (expβ = 0.599, *p* < 0.05). Children living in the richer and the richest households were also less likely to be stunted compared to those living in the poorest households. The ICC estimates from the final model show that ~ 10% of the variability in stunting is attributable to differences in neighbourhoods.

### Spatial regression models

Table 4 shows the result of the spatial autoregressive models. The OLS model shows that the percentage of uneducated mothers and the mean annual temperature are significantly associated with stunting among children aged under five. A percentage increase in the proportion of mothers with no formal education increases the percentage of stunted children by 0.343 (*p* < 0.05). A degree Celsius increase in the mean annual temperature reduces the percentage of stunted children by 3.056 (*p* < 0.01). The result of the Moran I test for the OLS means we can reject the hypothesis that the residuals from the model are not independent and identically distributed; that is, it indicates autocorrelation is present in the model. The spatial error model (SEM) similar to the OLS model shows that the percentage of uneducated mothers and district mean annual temperature are significantly associated with district percentage of stunted children. The spatial effect (λ = 0.497, *p* < 0.05) and the Wald test for the spatial term (*p* < 0.05) suggest the presence of significant spatial error dependence in the residuals. Unlike the OLS and SEM models, the spatial lag model (SAL) and spatial durbin error (SDEM) models' results are decomposed into direct effect (or within unit effect), indirect effect (across units effect or effect of neighbouring units), and total effect (the sum of the within and across units effects). In SAL and SDEM models, when a predictor is significantly associated with an outcome and the unit and across unit effects are in the same direction, a spillover effect is said to have occurred^41^. The spatial effect for the SAL model was not statistically significant and there was no spillover effect in the model. The SDEM model (Table S1) indicates that the spatial effects in the model are statistically significant for mean annual rainfall and the spatial error. Similar to the SAL model there were no spillover effects in the SDEM model. The model diagnostics for the spatial models show an improvement in the SDEM model compared to the OLS, SEM, and SAL models. The AIC value for the SDEM model was the lowest (AIC = 903.854) and the model accounted for ~ 33% of the variability in the percentage of stunted children (Pseudo R^2^ = 0.326).

**Table 4:** Spatial regression models for the percentage of stunted children by districts.

Predictor variables	OLS model	SEM model	SAL model	Direct	Indirect	Total
Uneducated mothers (%)	0.343 (0.140)*	0.347 (0.129)**	0.330 (0.137)*	0.330 (0.137)*	− 0.019 (0.041)	0.311 (0.144)*
Unemployed mothers (%)	0.073 (0.087)	0.055 (0.081)	0.075 (0.083)	0.075 (0.083)	− 0.004 (0.011)	0.071 (0.078)
Uneducated fathers (%)	− 0.241 (0.136)	− 0.229 (0.129)	− 0.233 (0.131)	− 0.233 (0.131)	0.014 (0.030)	− 0.219 (0.131)
Unemployed fathers (%)	0.139 (0.239)	0.156 (0.226)	0.147 (0.229)	0.148 (0.230)	− 0.009 (0.024)	0.139 (0.215)
Poorest and poorer households (%)	0.106 (0.069)	0.118 (0.066)	0.107 (0.066)	0.107 (0.066)	− 0.006 (0.014)	0.101 (0.063)
*Mean heads of livestock per km*^*2*^	0.052 (0.031)	0.047 (0.030)	0.053 (0.030)	0.053 (0.030)	− 0.003 (0.007)	0.050 (0.029)
*Mean aridity 2015 and 2010*	− 0.346 (0.465)	− 0.472 (0.485)	− 0.351 (0.444)	− 0.351 (0.444)	0.021 (0.052)	− 0.330 (0.419)
*Mean rainfall 2015 and 2010 (mm)*	0.008 (0.008)	0.014 (0.008)	0.007 (0.008)	0.007 (0.008)	− 0.001 (0.001)	0.007 (0.008)
*Mean diurnal temperature 2015 and 2010 (°C)*	− 4.324 (2.506)	− 4.459 (2.719)	− 4.407 (2.402)	− 4.410 (2.405)	0.259 (0.590)	− 4.151 (2.285)
*Mean annual temperature 2015 and 2010 (°C)*	− 3.056 (1.167)**	− 3.017 (1.240)*	− 3.083 (1.116)**	− 3.086 (1.118)**	0.181 (0.402)	− 2.904 (1.098)**
**Spatial effects**						
*Spatial error—λ*		0.497 (0.170)*				
*Spatial lag—ρ*			− 0.070 (0.160)			
**Model diagnostics**						
*Wald test for spatial term*		5.83*	0.89			
*Adjusted R-squared*	0.111	0.151	0.181			
*AIC*	907.074	905.321	910.199			
*Moran I*	4.130*					

Standard error in parenthesis; ***p < 0.001; **p < 0.01; *p < 0.05.

### Multi-scale geographically weighted regression

**Figure 2:**
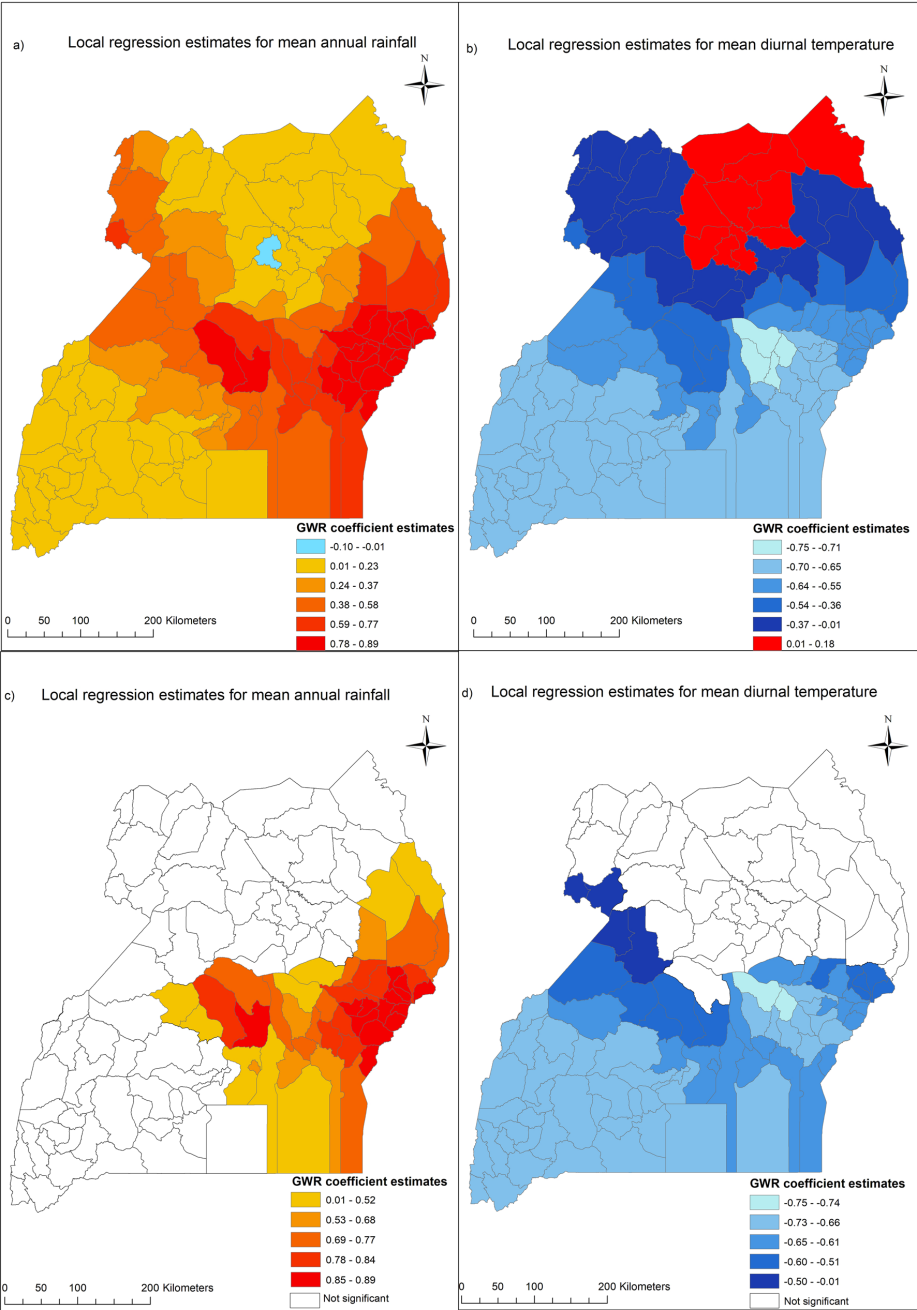
Multiscale geographically weighted regression local estimates for (**a**) mean annual rainfall, (**b**) mean diurnal temperature, (**c**) mean annual rainfall at *p* < 0.05 (statistically significant), and (**d**) mean diurnal temperature at *p* < 0.05 (statistically significant). Generated with ArcMap 10.6 by ESRI (https://desktop.arcgis.com/en/).

Table 5 displays the summary statistics of estimated coefficients of the local terms (MGWR model), as well as the optimal bandwidth for each predictor and the result of the Monte Carlo test of non-stationarity. The spatial heterogeneity test (Monte Carlo test of non-stationarity) shows a statistically significant result for mean annual rainfall and mean diurnal temperature (*p* < 0.05), thus suggesting spatial variability in the two variables. The model diagnostics show that the MGWR model improved significantly compared to the OLS model and the global spatial regression model. The MGWR model had the smallest AIC value (295.528) and the highest R^2^ value (0.462)—that is, the MGWR model explained ~ 46% of the variability in stunting rate among children aged under five. This indicates for our spatial models, MGWR is statistically preferable compared to the SEM, SAL, and SDEM models. Figure 2 shows the variation of the estimates for the local effects of mean annual rainfall and mean diurnal temperatures on the rate of stunting among children. With the exception of Kole district, an increase in mean rainfall was associated with an increased rate of stunting at the district level (Fig. 2a). The association was only statistically significant (*p* < 0.05) for some districts in the central and eastern parts of Uganda (Fig. 2c). The visualization of the local estimates for mean diurnal temperature showed mixed patterns. For the majority of the districts in Uganda, an increase in the district mean diurnal temperature was associated with a reduction in the rate of district-level stunting (Fig. 2b). However, in some districts in the northern part of the country (shown in red in Fig. 2b) increase in district mean diurnal temperature increased the rate of stunting among children. Figure 2d indicates this association was statistically significant (*p* < 0.05) for the districts in the southern parts of the country and a few districts in the northern-western and eastern parts of the country.

**Table 5:** Summary of the MGWR model with the optimal bandwidth and the Monte Carlo non-stationarity test result for the predictors.

Predictor variables	Mean (STD)	Minimum (maximum)	Median	Bandwidth	Non-stationarity (*p* values)
Uneducated mothers (%)	0.362 (0.115)	0.172 (0.488)	0.423	110	0.193
Unemployed mothers (%)	0.118 (0.059)	0.052 (0.216)	0.091	110	0.377
Uneducated fathers (%)	− 0.224 (0.051)	− 0.334 (− 0.161)	− 0.209	110	0.209
Unemployed fathers (%)	− 0.033 (0.024)	− 0.075 (0.022)	− 0.044	110	0.677
Poorest and poorer households (%)	0.386 (0.025)	0.349 (0.439)	0.388	110	0.750
*Mean heads of Livestocks per km2*	0.119 (0.010)	0.104 (0.152)	0.118	110	0.968
*Mean aridity 2015 & 2010*	− 0.168 (0.069)	− 0.267 (− 0.020)	− 0.182	110	0.080
*Mean rainfall 2015 & 2010 (mm)*	0.394 (0.312)	− 0.003 (0.893)	0.328	44	***0.033***
*Mean diurnal temperature 2015 & 2010 (°C)*	− 0.490 (0.261)	− 0.751 (0.183)	− 0.635	56	***0.018***
*Mean annual temperature 2015 & 2010 (°C)*	− 0.528 (0.053)	− 0.653 (− 0.453)	− 0.530	110	0.649
**Model diagnostics**					
*AIC*	295.528				
*AICc*	308.777				
*R-squared*	0.462				
*Adjusted R-squared*	0.325				

*p* values < 0.05 in bold.

## Discussion

Geographic concepts of spatial dependence and spatial heterogeneity are important in enhancing our understanding of the relationship between socioeconomic, climatic factors and childhood malnutrition. These concepts are based on the tenet that the interaction of socioeconomic and broader contextual factors with childhood malnutrition is not static across space. Likewise, socioeconomic and place-based vulnerabilities and their effect on health outcomes vary geographically, hence the need to explore the spatial relationships between individual factors and health outcomes, as well as that between broader contextual factors and health outcomes. The analytical approaches employed in this study enables us to address this crucial issue of spatial relationships or associations between socioeconomic, climatic factors and childhood malnutrition. The descriptive maps show districts in the northern and eastern parts of the country tend to be socioeconomically disadvantaged compared to the rest of the country. This is evident in the descriptive maps (Fig. 1) which show clustering of the district proportion of poor households the northern and eastern regions of Uganda; likewise, there is clustering of the district proportion of mothers with no formal education in the north-eastern part of Uganda. These regions have experienced decades armed conflicts, including the Lord’s Resistance Army insurgency, contributing to the vulnerabilities of women and children in these parts of Uganda^42,43^.

First, we employed multilevel modelling to account for neighbourhood and district variation (or contextual effects) in childhood malnutrition which cannot be account for in the normal regression approach^44^. Next, we used spatial regression models to examine spatial dependency in stunting and spatial heterogeneity in the effect of socioeconomic and climatic factors on the rate of stunting among children aged under five. The results of the multilevel analysis confirm significant between neighbourhood variations in stunting; and between district variation in stunting when we consider child, parents and household factors only. The spatial regression models suggest the error terms are correlated across districts, that is, neighbouring districts’ stunting influence a district’s rate of stunting due to unmeasured factors that are correlated across districts or systematic measurement error. The MGWR model also confirms spatial variation (heterogeneity) in the association of mean annual rainfall and mean diurnal temperature with the district rate of stunted children.

Consistent with the findings from existing studies, our multilevel model shows that climatic factors and other contextual factors are significantly associated with stunting among children. However, the direction of this association differs across the context of the study. In arid and semi-arid countries, results show that an increase in annual rainfall or precipitation reduces the likelihood of stunting among children aged under five^8,9,21,26,45^. An increase in the mean annual rainfall in arid and semi-arid countries sustains and enhances healthy food production, given the environmental and climatic conditions of these countries^8,21,26,45^. In Somalia, Kinyoki et al. (2016) observed that a mm increase in annual rainfall reduced the likelihood of children experiencing stunting by 12%. In contrast, Uganda is a tropical country with two distinct rainfall seasons and a mean annual rainfall of 1200 mm^46^. An excessive amount of rainfall has the potential to negatively affect agricultural output through flooding, potentially creating food insecurity in many households that depend on subsistence agriculture for nutritional needs^3,17,47^. The country’s humid condition means an optimal temperature or period of dryness is necessary for ensuring the harvesting of agricultural produce. Our finding shows that an increase in mean annual rainfall increases the likelihood of stunting; while an increase in mean annual temperature and mean diurnal temperature reduces the likelihood of stunting among children in the country. However, excessive dryness (drought and aridity) may also affect food production leading to food insecurity and resulting in cases of malnutrition among children. Our study also reveals that an increase in the mean head of livestock per km2 increases the likelihood of stunting. We argue that high-density livestock may potentially be indicative of commercial animal husbandry which does not necessarily translate into direct household consumption. Also, households in areas with a high density of livestock may have to compete with this commercial production activity for available arable lands^48^.

We also observed that child, parent and household characteristics are significantly associated with stunting. Children from poor and socioeconomic disadvantaged households are more likely to be stunted. The result shows children with below-average birth weight are also more likely to be stunted. These findings support evidence from existing studies. Research on childhood malnutrition in the sub-Saharan African sub-region shows that low birth weight, male sex, low paternal education and poor households are consistent risk factors for stunting and other indicators of malnutrition among children aged under five^31,32,34^. In Ghana, Novignon et al. (2015) observed that maternal primary education and secondary education account for 13 and 11%, respectively, of inequality in stunting among children. The findings of this study suggest that child, parents and household characteristics have a modifying effect on the association between some climatic factors and stunting. In the final multivariate multilevel analysis, drought episode and mean diurnal temperature, as well as, mean head of livestock per km^2^ became statistically significant after controlling for child, parent and household characteristics. Likewise, the statistical significance of some child, parent and household characteristics disappeared after controlling for contextual factors, including climatic conditions, in the final multivariate multilevel model.

The global multilevel models show mixed results for the association between climatic factors and stunting among children. The results of spatial regression models (SEM and SDEM) indicate that childhood malnutrition in a given district in Uganda is likely to be influenced by the proportion of unemployed mothers and the average rainfall in the neighbouring districts as well as other contextual factors not accounted for in our model. Women in neighbouring districts may share similar socioeconomic characteristics thus socioeconomic vulnerabilities of women in neighbouring districts may reflect the conditions of women in a district under consideration. As indicated earlier, women or mothers’ socioeconomic vulnerabilities including unemployment increases the risk of childhood malnutrition among children aged under 5 years in Uganda^31^. The MGWR model (with its spatial variation ability) provides a contextual insight into the observed associations between the contextual factors and childhood malnutrition. First, the MGWR model shows that mean annual rainfall is significantly associated with stunting in districts in central and eastern parts of Uganda. The magnitude and the direction of the association suggest in these districts an increase in mean annual rainfall increases the percentage of stunted children aged under five. A plausible explanation for the association is the proximity of these districts to Lake Victoria. Districts in the vicinity of Lake Victoria receive the highest amount of annual rainfall^49,50^; thus, an excessive amount of rainfall could disrupt agricultural activities and food production for individuals and households in these districts. It is also possible that the local topography of these districts makes food production sensitive to rainfall above the mean annual total. This also could account for the spatial lagged effect of rainfall observed in the SDEM model (Table S1). Trade between neighbouring districts means, the effect of rainfall on agricultural productivity in a given district can equally affect food security in neighbouring districts thus contributing to childhood malnutrition in these neighbouring districts. On the contrary, the MGWR estimate magnitude and direction show that an increase in mean diurnal temperature in districts in the southern, central and eastern parts of the country, as well as few districts in the north reduces the percentage of stunting among children. Given the high amount of rainfall in these parts, an optimal variation in temperature (including diurnal variation) may be necessary to ensure crop survival and good yields^51,52^.

The findings of our study ought to be considered given its limitations. Our study did not consider important environmental factors, including soil type, local vegetation and food production systems, which could potentially influence food insecurity and childhood malnutrition. Likewise, the list of the child, parent and household characteristics used in this study is not comprehensive. In our analysis, we could not consider factors such as feeding practices, and sociocultural practices of the local communities which are known to be associated with stunting and other malnutrition indicators. The UDHS data used in this study comes from a cross-sectional survey hence we cannot draw causal inference from our findings. Another major limitation of this study is the use of self-reported data, such as the use of mother recall for child’s birth weight in the absence of a written record. This data is subject to recall bias as respondents can overestimate or underestimate the actual birth weight. Estimates from the aggregated data may not be true representations of district level childhood malnutrition and socioeconomic indicators, especially for districts where fewer clusters were selected for the UDHS survey. Although we explored spatial variation in this study, our analysis did not consider temporal variation. Future studies could potentially explore spatial and temporal variation in childhood malnutrition to provide a longitudinal dimension of its relationship with environmental, climatic, child, parent and household factors.

## Conclusion

Notwithstanding the limitations of this study, the findings have vital implications for future research and policy. Food production and household food security in many sub-Saharan African countries are at risk due to sensitivity to climatic conditions. Many households in Uganda, like most sub-Saharan African countries, are dependent on subsistence agriculture for their sustenance. Thus, excessive wetness or dryness due to climatic change risk can affect household agricultural productivity and food security; exacerbating malnutrition among vulnerable populations in this part of the world, particularly children. Without adequate food and income from agricultural production, children are more likely to be exposed to prolonged nutritional deficiencies contributing to their risk of stunting. The findings also suggest improving maternal and household socioeconomic conditions minimise the likelihood of stunting among children under 5 years in Uganda. Thus, there is a need for policymakers and stakeholder to direct resources to improve women’s socioeconomic status, household socioeconomic conditions and to mitigate the effect of climate change on agricultural productivity in the country. The novel use of MGWR methodology in this study shows that this association is not static across Uganda. It shows that the effect of mean annual rainfall and mean diurnal temperature on stunting may be dependent on the local context. The results show which areas might be sensitive to variability in these climatic conditions in relation to childhood malnutrition. This information is necessary for designing intervention measures and frameworks for addressing the adverse effect of climate change on childhood malnutrition taking into account the local context.

## Data and methods

In this study, we used the 2016 Uganda Demographic and Health Survey (UDHS). The UDHS is a nationally representative cross-sectional survey of women aged 14–49 years and men aged 15 to 54. The primary focus of the UDHS is to generate reliable information on fertility, family planning, infant and child mortality, maternal and child health, and nutrition. The 2016 GDHS used an update frame from the 2014 Uganda National Population and Housing Census (NPHC) as its sample frame^53^. The survey followed a two-stage sampling design. The first stage of sampling entailed choosing enumerations areas (EAs) from the 2014 NPHC delineated EAs as its sample point or primary sampling units (PSU). In Uganda, an EA—similar to a census tract in other parts of the world—is a small geographic area that covers an average of 130 households. A total of 697 PSUs were randomly selected from a complete list of 78,462 PSU used in the 2014 population and housing survey. 162 EAs were selected from urban areas and 535 EAs selected from rural areas. The second stage of sampling involved the systematic selection of 20,791 households from the selected EAs (hereinafter called PSUs) or sampling clusters. The UDHS datasets include geographic data that contains point data with the GPS coordinates of sampled PSUs. These coordinates can be linked with the UDHS survey datasets, including the child recode dataset which contains information on birth history, health and anthropometric records of children born in the last 5 years (prior to the survey) to all women interviewed. Detailed information on the sampling and methods used in the 2014 GDHS is available in the final report.

The UDHS data was linked with the 2014 Uganda district GIS shapefile and all 697 PSUs were matched to their respective districts. The UDHS point data has information on the districts were the PSUs (clusters) were selected labelled “ADM1DHS” the attribute table; this information matches the PSUs to 112 districts (at the time of the 2014 NPHC). In this study, we overlaid the DHS point data shapefile over a 2014 district shapefile (containing 112 districts) to match the points to their respective districts using the Join Data function in ArcMap. With this function, one can link spatial datasets (in our case the DHS point data and the district shapefile) based on their spatial locations. The output file will contain information from both point data (PSUS) and the district shapefile including the longitudinal and latitudinal information of the districts. The procedure for linking the UDHS survey data with the output containing district data is described in details elsewhere^54^. UDHS survey protocol was reviewed and approved by the ICF Institutional Review Board (IRB) and an IRB in the host country. ICF IRB was to ensure that the survey complies with the U.S. Department of Health and Human Services regulations for the protection of human subjects (45 CFR 46), while the host country IRB ensures that the survey complies with laws and norms of the nation.

### Measures

#### Outcomes

Childhood malnutrition in this study was measured by stunting (height-for-age). In the DHS, height is measured with a Shorr Board measuring; children under 24 months were lying down while older children were measured standing^53^. Stunting, as an indicator of malnutrition, reflects a linear growth of a child and is influenced by long period deficiencies in calories and protein; that is, it reflects cumulated or long period malnutrition in children^55^. In line with the WHO convention, children in the UHDS sample were classified as stunting if their height-for-age z scores are below minus two standard deviations (< -2 SD). This outcome was also aggregated by districts for our spatial analysis. That is, the outcome for the spatial analysis was the percentage of children under age five that experienced stunting by districts.

#### Contextual factors

The contextual data also comes from the UDHS datasets. The UDHS datasets, like other demographic and health surveys supported by the U.S. Agency for International Development (USAID) and implemented by ICF, include geospatial covariates that contain environmental, climatic and geographic information on DHS clusters^56^. These geographic covariates can easily be linked with other datasets using the cluster codes. A detailed description of the data extraction processing can be found elsewhere^56^. In this study, the key contextual factors were: the average number of drought episodes, aridity index, average annual rainfall (in mm), average diurnal temperature (in °C), average annual temperature (in °C), and average heads of livestock (cattle, chickens, ducks, goats, pigs and sheep) per kilometres square. Drought episodes were categorized as 1(low) and 10(high). Aridity index ranging from 0(most arid) to 300(most wet) was defined as the ratio of annual precipitation to annual potential evapotranspiration^56^. Except for drought episodes, the mean values of all contextual factors were computed as the average for the years 2010 and 2015. These variables were not a continuous yearly measurement but measurement for discrete periods. For instance, the mean annual temperature variable in the UDHS geographic covariate datasets covers 2000, 2005, 2010, and 2015. Drought episode in the DHS was constructed based on precipitation data from 1985, 1990, 1995, 2000, 2005, 2010, and 2015. These variables were also aggregated by districts for the spatial analysis computed as the mean value of all sampled clusters or EAs in the district.

#### Child, parental and household factors

Based on the evidence from existing research, we included child, parental and household characteristics as predictors of childhood malnutrition. We employed sex and weight at birth as measures of child characteristics. Birth weight was obtained from either written record or mother’s recall (in the absence of a written record). The UDHS measure on a child’s weight at birth was an ordinal variable with the following response categories: very large, larger than average, average, and smaller than average. Parent’s socioeconomic characteristics were measured by the highest level of education and sector (or type of employment). In the UDHS, household wealth was constructed using data on household asset ownership. The respondents were categorized into five groups (or wealth index): richest, richer, middle, poorer, and poorest. The location of the household, urban or rural, was also included as a measure of household characteristics. Similar to the outcome and contextual factors, we computed the percentage of mothers with no formal education and fathers with no formal education by districts as predictors for the spatial analysis. The list of socioeconomic predictors for the spatial analysis also includes the percentage of poorest and poorer households by districts—defined in the spatial analysis as poor households.

### Analysis

We adopted three analytical techniques to understand the nature of the association between socioeconomic characteristics, climatic factors, and childhood malnutrition. First, we adopted a multilevel mixed-effect analysis to accommodate the hierarchical or stratified nature of the UDHS data. Using multilevel mixed-effect analysis, we can assess the effect of context or place variations via an assessment of the variance (or standard deviations) of the model parameters^57,58^. An initial assessment of our outcome variables indicated it was asymmetrically distributed hence we specified the complementary log–log (cloglog) link function. The complementary log–log link function relaxes the symmetrical assumption of logistic regression and it is ideal for events or outcomes with a very large or very small probability (incidence) of occurrence^59^. Using the logistic link function for such outcomes may produce biased parameter estimates. Three-level multilevel mixed-effect complementary log–log models were built with children nested in neighbourhoods (defined PSUs) and neighbourhoods nested in districts. First, we fitted a bivariate complementary log–log model to examine the association between indicators of childhood malnutrition and each of predictor variable included in our study. This was followed by three multivariate model multilevel mixed-effect complementary log–log models. Model 1 examined the association between contextual factors (including climatic factors) and childhood malnutrition. The second model (Model 2) focused on the association between child, parental, and household characteristics while the final model (Model 3) is a full model that includes both contextual variables and child, parental, and household variables.

The second analytical approach entailed fitting spatial regression models to assess spatial dependency. Three main spatial regression methods were employed to assess the relationship between district-level factors and child malnutrition, noting the role of contextual factors in the relationship may vary differently. Before fitting these models, we employed Moran I statistics to assess whether there is autocorrelation in the ordinary least square regression (OLS) model. A statistically significant result indicates that ignoring spatially lagged dependent or spatially lagged covariates or spatial error dependence in the OLS model will bias the estimates (parameter and standard error estimates) for the covariates in the model^40^. The first spatial regression model confined the spatial autocorrelation to the error term—that is, a spatial error model (SEM). In SEM model, we argue the childhood malnutrition is dependent on observed local characteristics and the error terms are correlated across space—that is, accounting for excluded spatial effects (effects not examined in the model) that could explain the observed spatial autocorrelation in the residuals^41,60^. The second model—spatial lag model (SAL)—confined the spatial autocorrelation to the outcome in neighbouring districts or spatial lags of the outcome variable. SAL is based on the assumption that childhood malnutrition in a given district is influenced by childhood malnutrition in neighbouring districts. It does not make any assumptions on the nature of the spatial relationship among contextual factors considered in the model and draws heavily on the spatial diffusion model or process^39,40^. In the final model (SDEM), we extend the spatial lag model be confining the spatial autocorrelation to spatial lag in the outcome and all predictors, and the error term^61,62^. That is, SDEM is based on the assumption that childhood malnutrition in a given district can be influenced by childhood malnutrition in neighbouring districts, contextual characteristics of neighbouring districts and other spatial effects not accounted for in the models.

A multi-scale geographically weighted regression (MGWR) was employed in the final analytical approach to assess spatial heterogeneity in the association between socioeconomic characteristics, climatic factors, and childhood malnutrition. Rather than providing an average global estimate for the relationship or association in the model as traditional regression (such as OLS) and global spatial regression (such as SEM, SAL and SDEM) models do, MGWR allows the model parameters to vary across the geographic units^63^. That is, the relationship between the predictor variables and childhood malnutrition is shown for each unit or district in the study. MGWR is an improvement of prior local statistical models—geographically weighted regression (GWR) and semi-parametric geographically weighted regression (SGWR). In both GWR and SGWR, the local association between an outcome and predictors are constrained to vary at the same spatial scale. These prior models were based on the assumption that the association(s) is influenced by processes operating at the same spatial scale^63^. The GWR model can be described as:

1 $${\text{y}}_{{\text{i}}} = \sum_{{\text{j}}}\upbeta _{{\text{j}}} \left( {\mu_{{\text{i}}} ,{\rm v}_{{\text{i}}} } \right){\text{x}}_{{{\text{ij}}}} +\upvarepsilon _{{\text{i}}} ,$$

where (μ_i_, v_i_) represent the coordinates for location i, x_ij_ is the jth predictor variable, βj(µ_i_,v_i_) is the jth coefficient, ε_i_ is the error term and y_i_ is the outcome variable. The SGWR model permits the existence of both global and local associations; the model is expressed as

2 $${\text{y}}_{{\text{i}}} = \sum_{{\text{j}}} {\text{a}}_{{\text{j}}} {\text{x}}_{{{\text{ij}}}} \left( {\text{a}} \right) + \sum_{{\text{l}}} {\text{b}}_{{\text{l}}} \left( {\upmu _{{\text{i}}} ,{\text{v}}_{{\text{i}}} } \right){\text{x}}_{{{\text{il}}}} \left( {\text{b}} \right) +\upvarepsilon _{{\text{i}}} ,$$

where yi, (μ_i_, v_i_) and ε_i_ are same as in model (1), a and b are the global and local predictor variables, respectively, a_j_ is the jth global coefficient, x_ij_(a) is the jth global predictor variable, x_il_(b) is the lth local predictor variable, b_l_(µ_i_,v_i_) is the lth local coefficient.

In contrast to GWR and SGWR, MGWR relaxes this assumption by allowing the associations between the outcome of interest and the predictors to vary at different spatial scale^63^. The MGWR model can be expressed as:

3 $${\text{y}}_{{\text{i}}} = \sum_{{\text{j}}}\upbeta _{{{\text{bwj}}}} \left( {\upmu _{{\text{i}}} ,{\text{v}}_{{\text{i}}} } \right){\text{x}}_{{{\text{ij}}}} +\upvarepsilon _{{\text{i}}} ,$$

where β_bwj_ is the calibration bandwidth for the jth conditional association, (μ_i_, v_i_), x_ij_, ε_i_ and y_i_ are the same as in the first formula (1).

In the MGWR model, we selected the bi-square weighting function as the adaptive kernel to account for the differences in the size of the districts and their varying population density^54,64^. The Golden Section search option was used for the bandwidth searching. This option successively narrows the range of values for the optimal bandwidth and returns the lowest score by comparing the optimization score for each model^64,65^. We used the corrected Akaike's Information Criteria (AICc) for the optimization criteria where the bandwidth the lowest AICc is selected and used in the analysis. As a model diagnostics technique, AICc accounts for the model complexity and also enables a comparison of the global model (OLS) and the local model (MGWR) to determine whether using spatial varying model improves the model^66,67^. The Monte Carlo test of spatial variability was specified to determine if the model parameters for the predictors significantly varied across the geographic units (districts). Reported *p* values less than 0.05 suggest spatial variability in the local term(s) or predictor(s). Natural breaks (Jenks) classification method was used classify the visualised descriptive data and local statistics (MGWR) results. Jenks classifies the data based on natural groupings inherent in the data. The unit of analysis for the spatial models was district or county (n = 112). The descriptive, multilevel analysis and spatial autoregressive statistical analyses were performed using STATA statistical software package version 16 by StataCorp (College Station, TX). We report the exponentiated coefficients (expβ) for the bivariate and multivariate (multilevel) regression results; while the spatial models report the beta coefficients. MGWR analysis was conducted in MGWR 2.1 software and visualised in ArcMap 10.6 by ESRI.

### Ethical approval and consent to participate

The data for this study was obtained from the Demographic and Health Survey (DHS) platform. Procedures and questionnaires for standard DHS surveys have been reviewed and approved by the ICF Institutional Review Board (IRB). Additionally, country-specific DHS survey protocols are reviewed by the ICF IRB and typically by an IRB in the host country. ICF IRB ensures that the survey complies with the U.S. Department of Health and Human Services regulations for the protection of human subjects (45 CFR 46), while the host country IRB ensures that the survey complies with laws and norms of the nation.

#### Informed and Voluntary Participation

Before each interview or biomarker test is conducted, an informed consent statement is read to the respondent, who may accept or decline to participate. A parent or guardian must provide consent before participation by a child or adolescent. DHS informed consent statements provide details regarding:

- The purpose of the interview/test
- The expected duration of the interview
- Interview/test procedures
- Potential risks to the respondent
- Potential benefits to the respondent
- Contact information for a person who can provide the respondent with more information about the interview/test

Most importantly, the informed consent statement emphasizes that participation is voluntary; that the respondent may refuse to answer any question, decline any biomarker test, or terminate participation at any time; and that the respondent's identity and information will be kept strictly confidential.

## Supplementary information

Supplementary Table S1.

## Data Availability

We do not have permission to share the research data. The main research data can be assessed through the DHS Program web portal. However, the data on the estimates of predictors and outcomes are available upon reasonable request.
