# Supplementary material for: Multilevel and spatial analyses of childhood malnutrition in Uganda: examining individual and contextual factors
Source: Sci Rep. 2020 Nov 18;10:20019. doi: 10.1038/s41598-020-76856-y (PMC7676238; doi:10.1038/s41598-020-76856-y)
Supplement: Supplementary file 1 — Supplementary Table S1. [file 41598_2020_76856_MOESM1_ESM.docx]

Multilevel and spatial analyses of childhood malnutrition in Uganda: Examining individual and contextual factors

Prince M. Amegbor^ab^*, Zhaoxi Zhang^ab^, Rikke Dalgaard^ab^ & Clive E. Sabel^ab^

^a^ Big Data Centre for Environment and Health (BERTHA)

Aarhus University,

DK-4000 Roskilde, Denmark

^b^ Department of Environmental Science,

Aarhus University,

Frederiksborgvej 399, DK-4000 Roskilde

Denmark

Correspondence to: Prince Michael Amegbor, BERTHA, Department of Environmental Science, Aarhus University, Frederiksborgvej 399, DK-4000 Roskilde, Denmark. Email: [pma@envs.au.](mailto:pma@envs.au.)dk

| Table S1. Spatial autoregressive model with spatial lag in the outcome, predictors, and the error term. | | | | |
| --- | --- | --- | --- | --- |
|  | SAR Model | Direct | Indirect | Total |
| Uneducated Mothers (%) | 0.245 (0.129) | 0.251 (0.126)* | -0.241 (0.361) | 0.010 (0.407) |
| Unemployed Mothers (%) | 0.097 (0.081) | 0.088 (0.079) | 0.370 (0.196) | 0.458 (0.229)* |
| Uneducated Fathers (%) | -0.259 (0.129)* | -0.262 (0.126)* | 0.121 (0.309) | -0.140 (0.343) |
| Unemployed Fathers (%) | 0.220 (0.230) | 0.243 (0.229) | -0.977 (0.596) | -0.735 (0.676) |
| Poorest and Poorer Households (%) | 0.189 (0.063)** | 0.188 (0.064)** | 0.043 (0.157) | 0.231 (0.164) |
| *Mean heads of Livestock per km2* | 0.053 (0.030) | 0.052 (0.029) | 0.051 (0.062) | 0.103 (0.068) |
| *Mean Aridity 2015 & 2010* | -1.559 (0.562)** | -1.576 (0.573)** | 0.735 (0.793) | -0.842 (0.855) |
| *Mean Rainfall 2015 & 2010 (mm)* | 0.038 (0.011)*** | 0.040 (0.011)*** | -0.062 (0.018)*** | -0.022 (0.016) |
| *Mean Diurnal Temperature 2015 & 2010 (°C)* | -5.306 (3.969) | -5.425 (4.099) | 5.128 (4.046) | -0.297 (3.465) |
| *Mean Annual Temperature 2015 & 2010 (°C)* | -3.579 (1.444)* | -3.584 (1.467)* | 0.215 (1.776) | -3.370 (1.670)* |
|  |  |  |  |  |
| **Spatial Effects** |  | | | |
| Uneducated Mothers (%) | -0.268 (0.455) | | | |
| Unemployed Mothers (%) | 0.472 (0.234)* | | | |
| Uneducated Fathers (%) | 0.118 (0.392) | | | |
| Unemployed Fathers (%) | -1.186 (0.722) | | | |
| Poorest and Poorer Households (%) | 0.077 (0.193) | | | |
| *Mean heads of Livestock per km2* | 0.070 (0.079) | | | |
| *Mean Aridity 2015 & 2010* | 0.712 (0.915) | | | |
| *Mean Rainfall 2015 & 2010 (mm)* | -0.072 (0.021)*** | | | |
| *Mean Diurnal Temperature 2015 & 2010 (°C)* | 5.689 (4.699) | | | |
| *Mean Annual Temperature 2015 & 2010 (°C)* | -0.192 (1.831) | | | |
| *Spatial Error - λ* | 0.5554 (0.235)* | | | |
| *Spatial lag - ρ* | -0.128 (0.301) | | | |
| **Model Diagnostics** |  | | | |
| AIC | 903.854 | | | |
| *Pseudo R-squared* | 0.326 | | | |
| *Wald test of spatial term* | 32.87*** | | | |
| standard error in parenthesis; ***p<0.001, **p<0.01, *p<0.05 | | | | |
